# Clinical implications of the American Joint Committee on Cancer (AJCC) 8th edition update in seminoma pT1 subclassification

**DOI:** 10.1186/s12894-020-00682-7

**Published:** 2020-08-20

**Authors:** Mário Fontes-Sousa, João Lobo, Helena Magalhães, João Cassis, Mariana Malheiro, Sância Ramos, Rui Henrique, Ana Martins, Maria Joaquina Maurício

**Affiliations:** 1 Serviço de Oncologia Médica, Centro Hospitalar Lisboa Ocidental, Estr. Forte do Alto Duque, 1449-005 Lisbon, Portugal; 2 Serviço de Anatomia Patológica, Instituto Português de Oncologia do Porto, Porto and Grupo de Epigenética e Biologia do Cancro (GEBC), Centro de Investigação do Instituto Português de Oncologia do Porto (CI-IPOP) e Instituto de Ciências Biomédicas Abel Salazar, Universidade do Porto (ICBAS-UP), Porto, Portugal; 3 Serviço de Oncologia Médica, Unidade Local de Saúde de Matosinhos (Hospital Pedro Hispano), Porto, Portugal; 4 Serviço de Anatomia Patológica, Centro Hospitalar Lisboa Ocidental, Lisbon, Portugal; 5 Serviço de Oncologia Médica, Instituto Português de Oncologia do Porto, Porto, Portugal

**Keywords:** Germ cell tumors, Seminoma, Cancer staging, AJCC, *Rete testis*, Biomarkers

## Abstract

**Background:**

Seminoma accounts for 30–50% of testicular germ cell tumors (TGCT)—the most common solid malignancy in men aged 15–35 years. The American Joint Committee on Cancer (AJCC) 8th edition (2018) created the subclassifications pT1a (tumor size < 3 cm) and pT1b (≥ 3 cm), despite not being universally recognized. *Rete testis* invasion (RTI) and tumor size > 4 cm are considered features associated with a higher recurrence risk, but not formally used for staging. The authors propose further understanding the subclassification’s potential impact in clinical practice, by summarizing current evidence and reviewing clinical cases in their institutions.

**Methods:**

All consecutive cases of seminoma stage I, pT1 treated in two institutions between January 2005 and December 2016 were included. Clinical data were retrieved, and variables were analyzed using SPSS. Relevant literature on the topic was reviewed.

**Results:**

Seminoma pT1 was identified in 58 patients. By using newly AJCC criteria, 29 (50%) would have been staged as pT1a and 29 (50%) pT1b. Median age at diagnosis was similar (33 in pT1a vs 32 in pT1b). Median follow-up time 5.8 years. Almost half (45%) of pT1b patients had a tumor size < 4 cm. The majority of either pT1a or pT1b were treated with chemotherapy or radiotherapy, reflecting more intensive approaches in the past. Three retroperitoneal recurrences were recorded (two in pT1a, one in pT1b, all under surveillance protocol); no deaths occurred. RTI and extensive necrosis (EN) were associated with pT1b (*P* <  0.0001 and *P* = 0.023, respectively), known adverse biological features.

**Conclusions:**

In our population, the exploratory analysis of the newly created AJCC criteria showed no significant difference in recurrence or death, although pT1b was associated with adverse biomarkers, such as RTI and EN, but its clinical relevance remains incompletely understood. Our results confirm an excellent prognosis, regardless of subcategorization, thus a larger population and a longer follow-up time are needed to understand prospectively the impact of the recently updated criteria. We would recommend using the latest AJCC staging system, although the individual risk of relapse, long-term toxicities and patient preferences should be taken into account when considering surveillance or active treatment adjuvant options.

## Background

**Fig. 1:**
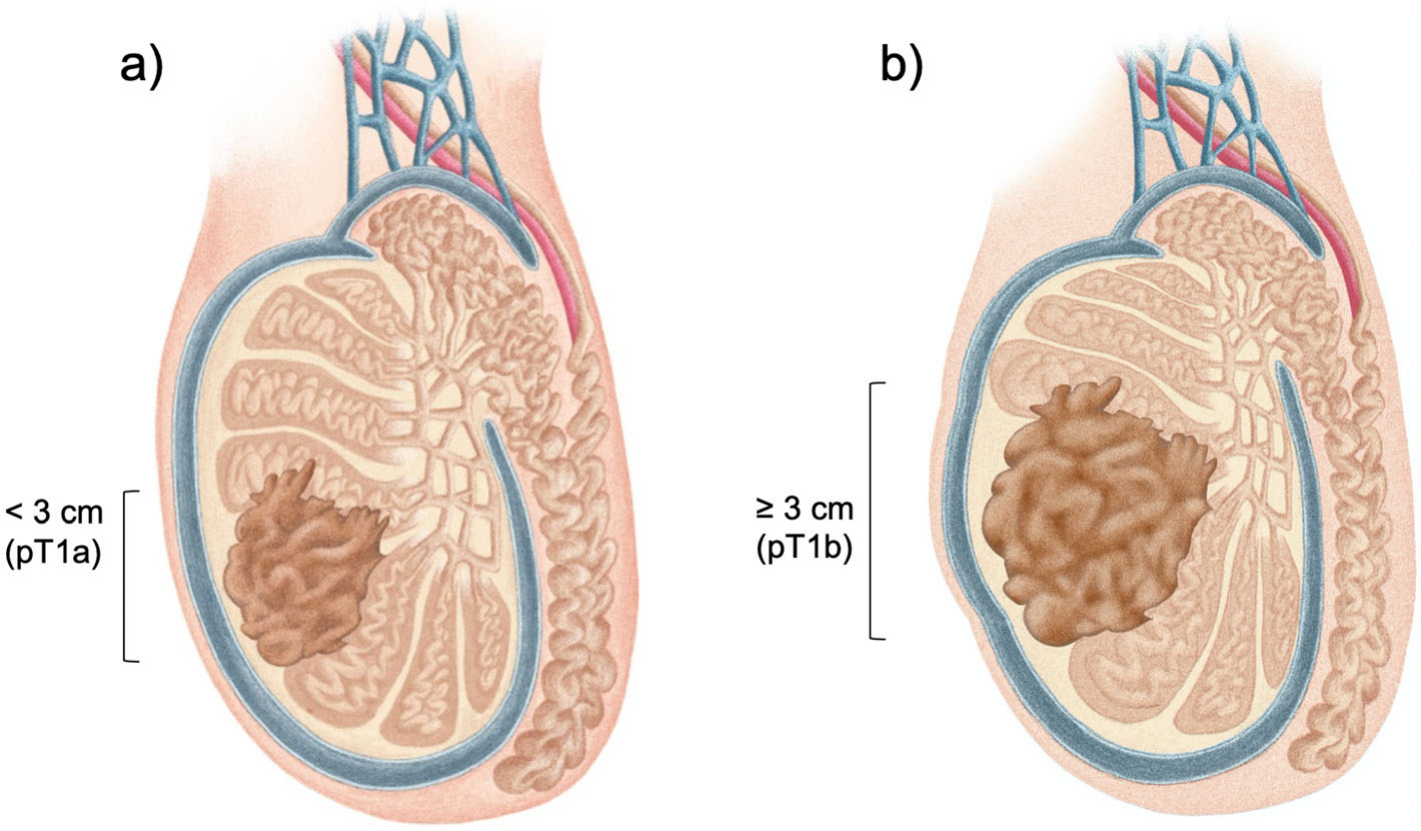
Newly implemented AJCC 8th edition exclusively for pT1 stage seminoma (**a**) tumor size < 3 cm (pT1a) and (**b**) tumor size ≥3 cm (pT1b). Note: these illustrations were created solely for the purpose of this article (by Inês Teixeira)

**Fig. 2:**
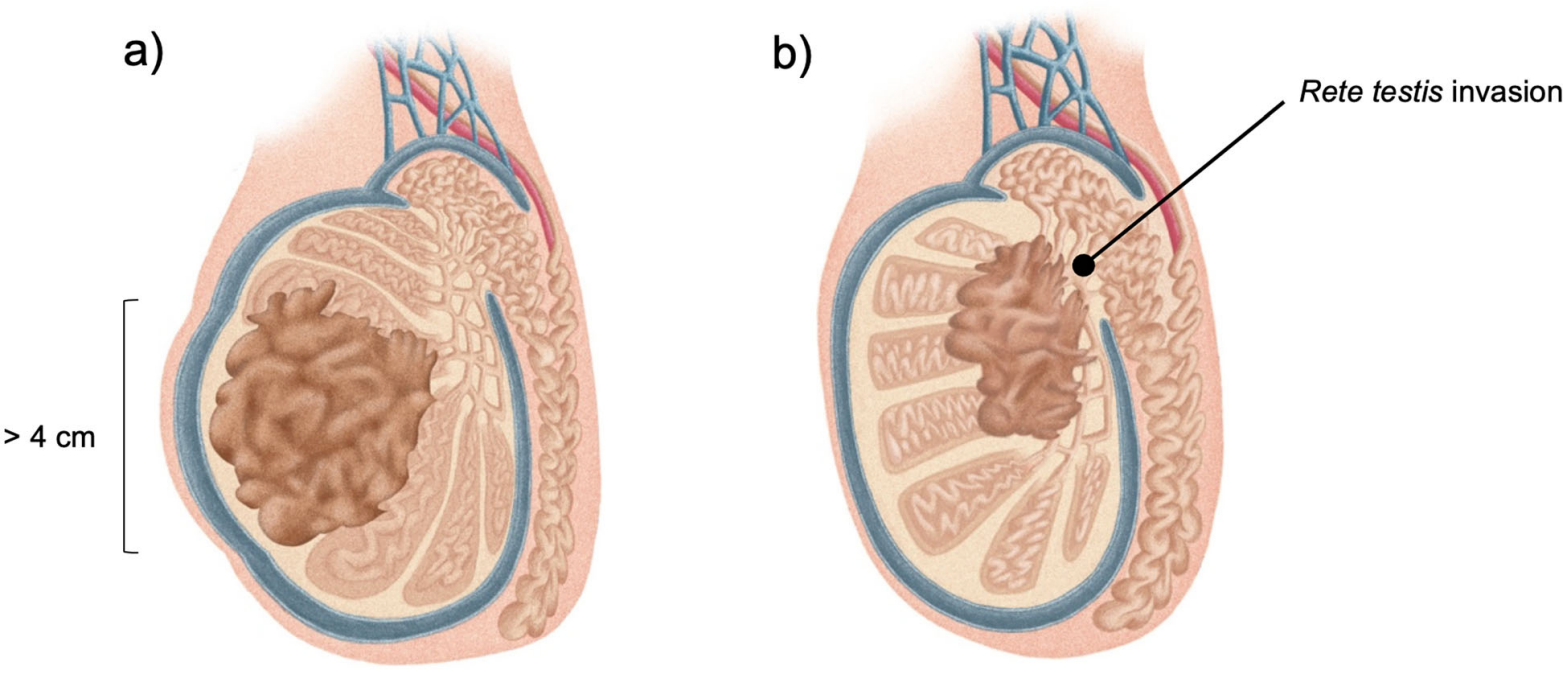
Classically described risk factors for seminoma: (**a**) tumor size > 4 cm and (**b**) *rete testis* invasion (RTI). Note: These illustrations were created solely for the purpose of this article (by Inês Teixeira).

Testicular germ-cell tumors (TGCT) are the most common solid malignancy in men aged 15-35 years old [1] and are classically divided in seminoma or non- seminoma. Seminoma histology accounts for 30-50% of the cases. TGCT are staged using the TNM(S) system, whose criteria is used worldwide, according to mostly overlapping *American Joint Committee on Cancer* (AJCC) and *Union for International Cancer Control* (UICC) manuals [2, 3]. Seminoma category pT1 is a tumor pathologically limited to the testis with no lymphovascular invasion (LVI) [2, 3]. Since the last updated edition (8^th^, 2017/18) [2], there has been divergence regarding seminoma’s pT1 category: AJCC created the subclassification of T1a (tumor < 3 cm) and T1b (tumor ≥ 3 cm)—Fig. 1 a and b, respectively, while UICC remained unchanged from the 7^th^ edition [3] (i.e. no subclassification). Of note, the subclassification does not change the stage grouping [2].TGCT have remarkably high cure rates, even at recurrence. Therefore, much focus has been placed in adjuvant treatment options. Specifically, when considering the importance of long-term toxicities management in patient selection. The adjuvant stage-specific treatment options in stage I seminoma include non-active treatment (surveillance) or active treatment, namely chemotherapy (CT) or radiotherapy (RT). In the clinical practice, a risk-adapted approach can be considered using historically adverse prognostic factors for stage I seminoma [4]: tumor size > 4 cm and *rete testis* invasion (RTI)—Fig. 2 a and b, respectively. These factors, albeit retrospectively identified, have been considered recurrence predictors [5]. So far, they have not been validated prospectively, except that in the absence of both of them, it constituted an indication of low recurrence rate (6%) [6] and therefore the evidence for its routine use in clinical practice is limited (in patients undergoing surveillance) [7]. Additionally, other factors should be considered for treatment decision, such as, patient preference or expected compliance with recommended follow-up protocols. An emphasis on tumor size has been of importance for a considerable time, since, for example, TGCT are not graded (thus, no clinical impact from its evaluation) and tumor markers will not be elevated in most cases (alpha-fetoprotein is never elevated in pure seminoma and human chorionic gonadotrophin may be elevated only in up to 30% of cases) [4].

## Objectives

We propose to retrospectively evaluate the impact of the AJCC 8th edition recent subclassification in stage I seminoma. Therefore, we aim to understand its potential use for prognosis and clinical decision, namely adjuvant treatment decision and follow-up protocol, by applying the current criteria in our population and making considerations on how these changes could impact the clinical practice.

## Methods

All consecutive cases of TGCT treated in two institutions (oncological center and a general hospital, located in Porto and Lisbon, respectively) were included, between January 2005 and December 2016, limited to seminoma Stage I. Clinical data were retrieved and re-reviewed according to most recent staging systems.

Pathology was also entirely re-reviewed by TGCT-dedicated Pathologists and updated according to the most recent 2016 World Health Organization (WHO) classification. The pathological criteria have been applied according to what was previously published [8]. In summary, size reflected the dominant tumor nodule in case of multifocality, as recommended in staging systems. Extensive necrosis (EN) was defined as “easily spotted on low power magnification, including geographic necrosis and contiguous areas of necrosis, including infarct-type necrosis”, as opposed to focal necrosis (“only spotted at high power magnification, often of isolated cells/cell nests”). *Rete testis* invasion (RTI) was documented when true stromal invasion was depicted, as indicated by the International Society of Urological Pathology (ISUP) recommendations [9]. Pagetoid extension was reported separately, but not counted as true *rete testis* invasion, as recommended.

The variables were analyzed using SPSS v.25. Potential statistical associations between categorical variables were evaluated using Chi-square test, using the two-sided Fisher’s significance level *p* <  0.05. Distribution of continuous variables among groups was assessed by the non-parametric Mann-Whitney U test.

The study was approved by the local Ethics board.

## Results

The population variables are characterized in Table 1. A total of 58 patients undergoing orchiectomy, no nodal or distant metastasis and diagnosed with pT1 seminoma were included, 29 of which would have been classified as pT1a (50.0%) vs 29 patients that would have been pT1b (50.0%), for the specified time frame (~ 11 years). The median follow-up time was 69 months, or approximately 5.8 years.

**Table 1:** studied clinical and pathological variables in seminoma Stage I cases. Additionally, cases are presented as aggregate and per center (Center 1 = Portuguese Institute of Oncology of Porto; Center 2 = Centro Hospitalar de Lisboa Ocidental)

	pT1 (*N* = 58)		*P value*
	pT1a (center 1) (center 2)	pT1b (center 1) (center 2)	
N (%)	29 (50%) (21)(8)	29 (50%) (19)(10)	–
Median Age at Diagnosis, years (min – max)	33 (17–52) (33)(33)	32 (21–66) (31)(36)	0.641
Median tumor size (cm)	1.7 (1.7)(1.8)	4.5 (4.3)(4.95)	–
Min – max size (cm)	0.7–2.8	3.0–12.0	
Tumor > 4 cm	-	16 (55.2%) (9)(7)	
*Rete testis* invasion (RTI)	2 (6.9%) (2)(0)	12 (41.4%) (9)(3/4^a^)	**< 0.0001**
Median Mitosis/10 HPF	10 (10)(^a^)	18 (18)(^a^)	0.098
Extensive Necrosis (EN)	12 (41.4%) (9)(3/5^a^)	22 (75.9%) (17)(5/9^a^)	**0.023**
Anaplastic features	12 (41.4%) (10)(2/5^a^)	14 (48.3%) (12)(2/9^a^)	0.793
Adjuvant Treatment			–
Surveillance	11 (44.0%)	6 (27.3%)	
Active treatment	14 (56.0%)	16 (72.7%)	
*CT*	*2 (8.0%)*	*3 (13.6%)*	
*RT*	*12 (48.0%)*	*13 (59.1%)*	
Testicular contra-lateral metachronous tumor	1 (5.3%)	0	–
Distant Recurrence^b^	2 (0)(2)	1 (0)(1)	–
Cancer-specific death	0	0	–

Abbreviations: *CT* Chemotherapy, *EN* Extensive Necrosis, *RT* Radiotherapy, *RTI Rete testis* invasion, *HPF* High-Power Field.

Notes: sizes are presented in cm as is AJCC; *P* values in bold indicate statistical significance.

^a^Data missing; ^b^ all distant recurrences were in the retroperitoneum

The median age at diagnosis was similar between groups (33 years in pT1a vs 32 years in pT1b, *P* = 0.641).

In pT1a patients the median tumor size was 1.7 cm (0.7–2.8 cm) vs 4.5 cm (3.0–12.0 cm) in pT1b patients. In the latter subcategory, 16 cases (55.2%) were > 4 cm (classically a ‘higher risk’ feature in seminoma). Therefore, 13 patients (44.8%) had their risk status ‘upscaled’, i.e. have a tumor size inferior to 4 cm, a classical ‘lower risk’ feature, but are now considered in the ‘higher risk’ pT1b category.

Four pathological features were evaluated: *rete testis* invasion, median number of mitosis/10 high power fields (HPF), evidence of EN and anaplastic features (Table 1). RTI and EN were significantly associated with pT1b tumors (*P* < 0.0001 and *P* = 0.023, respectively).

In pT1a patients, active treatment was delivered in 14 patients (48.3%) vs 16 patients (55.1%) in pT1b, being RT the predominant option in both subcategories. The adjuvant chemotherapy used was a single cycle of Carboplatin AUC 7; no data regarding RT dose or duration.

In one pT1a patient, a contra-lateral metachronous tumor was detected during follow-up (orchiectomy followed by surveillance protocol).

During follow-up, 3 cases of distant recurrence (retroperitoneal) were identified: 2 cases in pT1a (6.9%), neither initially with RTI, and 1 case in pT1b (3.4%), with both RTI and tumor size > 4 cm. All patients underwent BEP (cisplatin, etoposide and bleomycin) chemotherapy regimen, and currently have no evidence of disease.

The 5-year overall survival was 100% for both groups. No deaths were recorded during the follow-up period.

## Discussion

### Impact on prognosis and clinical decision

A focus on seminoma stage I is relevant since it is the most common single stage or histology of TGCT—it may account for up to 80% of seminomas and 40% of all testicular cancers [10]. Thus, having two staging criteria may have implications in clinical practice or clinical trial design: should we embrace the changes (AJCC) or ignore them (UICC)? Should adjuvant treatment be decided according to staging subcategory (i.e. higher risk could mean more aggressive treatment)? Should follow-up protocols take the new subcategories into consideration and have more intensive schedules in higher risk patients, even though, ultimately, survival might be similar? These clinically meaningful questions remain unanswered, creating additional anxiety on patients, their family and their physicians.

A fundamental notion is that the overall prognosis in stage I seminoma is exceptionally good [4]—confirmed in our retrospective analysis. Only few recurrences were recorded, and the 5-year overall survival rate was 100%, regardless of subcategorization, with a median follow-up time ~ 6 years. The occurrence of few events may be a limitation (i.e. low rate of recurrence or death), indicating high curability rate even after recurrence. This remarkable prognosis, plus low incidence, characteristically lead to accrual failure in TGCT clinical trials [11]. Retrospective data have emerged suggesting the 3 cm cut-off was significantly associated with metastatic status at presentation, but only if LVI or spermatic cord invasion (SCI) were present [12], which are known independent high-risk features. This conclusion is not applicable to pT1 stage, since LVI is, per definition, at least pT2, and SCI is pT3 [2, 3]. The three recurrences identified (3 out of 17 surveillance patients, 17.6%), are within the overall estimation of 15–20% risk of recurrence for stage I seminoma without adjuvant treatment [13].

Another clinical concern is adjuvant treatment selection. As previously mentioned, the presence of RTI and tumor size > 4 cm can be taken into account in order to establish an individual recurrence risk and help indicate treatment over surveillance [4]. Interestingly, size ≥3 cm (the current AJCC pT1b subcategory cut-off) was significantly associated with RTI (*P* < 0.0001) and EN (*P* = 0.023) in the univariate analysis. This may indicate that increasing size is related to adverse pathological features, a generally coherent finding, and yet, its clinical relevance is unknown at this time and should be a focus of future research. Additionally, two recurrences were identified in the pT1a group (vs 1 in pT1b), with neither case showing initially RTI, which underscores the need to have better biomarkers to predict recurrence (in seemingly classical low risk patients).

Some considerations are justified regarding patients with a tumor size ≥3 cm but ≤4 cm, which were almost half of the pT1b group (44.8%, Table 1). Following the 8th edition AJCC criteria, they would be considered at higher risk of recurrence (vs pT1a) and yet below the classically considered higher risk size of 4 cm. Observing the combinations of no RTI/RTI and tumor size ≤4 cm and > 4 cm (Table 2) one can realize that even within the pT1b category, different risk groups can be identified. We show it is a very heterogenous group. This is in accordance with a nomogram that suggested risks of recurrence depending on how many risk factors were present: 12% risk of recurrence (if none present), 16% (presence of either one), and 32% (in the presence of both) [14]. These data indicate that we need more reliable prognostic biomarkers (the absence of both represent a non-negligible 12% recurrence risk), as stressed previously. On the contrary, being both factors together, the risk is double than being just one present. This suggests that almost 1/3 of patients will recur, reinforcing RTI prognostic role along with size. RTI is explicitly considered by AJCC as a pathological feature that does not change staging (based on large contemporary cohorts data) [2]. Thus, the clinical significance of RTI remains controversial [7]. Combining our experience, in particular and as mentioned before, the observed significant association between RTI and increased tumor size (*P* < 0.0001, Table 1), with large retrospective data whose importance is recognized in international guidelines [4, 13], RTI may still have a role in clinical practice. Actually, RTI could be, in the future, included for staging purposes as an adverse feature within the pT1b subclassification, such as a suffix pT1b(0) for no RTI (lower risk) vs pT1b(1) for RTI (higher risk)—like the precedent in melanoma M1 disease staging with (0) indicating normal lactate dehydrogenase (LDH) and (1) indicating elevated LDH [2]. This change could enable the evaluation of its significance prospectively, essentially like the decision to create pT1a vs pT1b subclassifications based on a size cut-off.

**Table 2:** distribution within the pT1b group (tumor size ≥3 cm) of classically defined high risk features in stage I seminoma (RTI and tumor size > 4 cm – Fig. 2 a) and b), respectively), that are not formally part of the staging criteria, but are frequently used to guide clinical decision regarding surveillance vs adjuvant treatment

pT1b (tumor size ≥3 cm)			
Variables	Tumor size ≤4 cm	Tumor size > 4 cm	Total
No RTI	**4 (18.2%)** (lower risk category)	6 (27.3%)	10
RTI	7 (31.8%)	**5 (22.7%)**^**a**^ (higher risk category)	12
Total	11	11	22^b^

Abbreviation: *RTI Rete testis* invasion.

^a^ the patient that recurred in the pT1b group had RTI plus tumor size > 4 cm, thus within the higher risk category.

^b^ Data missing in 7 patients

### Analysis of potential bias

This is an exploratory retrospective analysis of current criteria applied to a population that was treated regardless of them. Therefore, the interpretation of results is considerably limited, although it may offer glimpses into future directions, namely clinical or basic research aims of focus and unmet needs.

The follow-up time may be insufficient to detect enough events (recurrence or death) in a small population, which is a limitation in stage I seminoma studies, as previously detailed [15]. Nevertheless, the study that suggested the 3 cm cut-off (and now the basis for seminomas’ pT1a vs pT1b cut-off in AJCC’s) used the 3-year recurrence risk endpoint [16], thus considering that our population has over 5 years of follow-up we deemed appropriate to proceed with the analysis. We focused on stage I seminoma in order to obtain a more homogenous population, risking a smaller sample.

Our population was treated from 2005 onwards, and at that time one of the popular adjuvant treatment options was RT, which could explain the notable percentage of treated patients with this technique (43.1% of the population, vs 8.6% chemotherapy and 29.3% surveillance), and might also help explain the few recurrences, although, retrospectively, some patients may have been over- treated according to current trends of thinking [17]. The patients that recurred in the retroperitoneum (2 in pT1a group and 1 in pT1b group, all under surveillance protocols) were effectively treated, and their current status is no evidence of disease.

Additionally, some pathological data were missing due to suboptimal evaluation conditions of the material under analysis. Despite great focus being rightly placed on late toxicities, we were not able to gather meaningful clinical data regarding these issues.

## Conclusions

Incorporating ‘classical’ risk factors for recurrence with the new seminoma pT1 subgrouping by newly created AJCC criteria may pose new challenges in clinical practice. In our population, a two-center exploratory analysis, showed no difference in recurrence or death, although pT1b was significantly associated with adverse pathological findings, such as RTI and EN. This was an exercise to understand our population regarding recent staging changes, that are not common to both systems in use. Our main goals were to review this pressing topic, share and discuss our daily practice concerns and propose ways of addressing this issue. A larger population and a longer prospective follow-up time are needed to understand the impact of the updated criteria, namely when considering clinical trial design, disease prognosis, adjuvant treatment options and tailored follow-up protocols for the individual patient. Until then, we would recommend using the AJCC staging system, while recognizing that tumor size seems to matter in regard to seminoma, the individual risk of relapse, long-term toxicities and patient preferences should be taken into account when considering surveillance or active treatment options.

## Data Availability

The datasets used and/or analyzed during the current study are available from the corresponding author on reasonable written request.

## Abbreviations

AJCC: American Joint Committee on Cancer
CT: Chemotherapy
EN: Extensive necrosis
HPF: High power fields
ISUP: International Society of Urological Pathology
LDH: Lactate dehydrogenase
LVI: Lymphovascular invasion
RT: Radiotherapy
RTI: *Rete testis* invasion
SCI: Spermatic cord invasion
SPSS: Statistical Package for the Social Sciences
TGCT: Testicular germ-cell tumors
UICC: Union for International Cancer Control
WHO: World Health Organization
